# Supplementary material for: Invariance in ecological pattern
Source: F1000Res. 2019 Dec 12;8:2093. [Version 1] doi: 10.12688/f1000research.21586.1 (PMC7008606; doi:10.12688/f1000research.21586.1)
Supplement: Peer review history

# Peer review: Reviewer response for version 1

**DOI:** 10.5256/f1000research.23789.r57848

**Published:** 2020-02-07

**Authors:** Neil McRoberts

**Affiliations:** 1 Department of Plant Pathology, University of California, Davis, Davis, CA, USA

This paper by Frank and Bascompte explores ideas about the origins of statistical pattern in biology developed in previous work by Frank and Frank & Smith, and applies them to the question of the distributions of species population sizes commonly observed in community ecology. As the authors note, the distribution of population sizes in a given ecological community will tend to follow quite closely one of two probability distributions – the logarithmic or the skewed log-normal – irrespective of the physical size of the organisms in the community of interest, and the number of species involved. Why this should be is an important question, and one that points toward some general rule or principle of ecology that would be fascinating and potentially valuable to understand. In providing their own explanation, the authors follow a formula proven by Frank in earlier publications: The problem is outlined, previous efforts to explain it are described, noting *ad hoc* or special features which limit their generality, before the authors’ own explanation is described and its relative merits compared with the earlier attempts.

 It is worth taking a moment to put this work in a brief historical context of the more general topic of research that has occupied Frank for more than a decade, because that broader (longer?) perspective says something useful about the process of research into theoretical biology and the development of theory. Frank’s earliest publications on the topic of probability patterns in nature were built around the idea that constraints on information contained in myriad stochastic processes, together with a principle of maximum entropy, leads to a few probability patterns being common. This explanation raises the question of how the constraints occur. Frank’s earlier work led to some principles to answer that question by assuming that the constraints arise in solving the maximum entropy expression for probability patterns with the method of Langrangian multipliers. Of course, since what’s needed is an explanation for empirical observation, none of this is supposed to be happening to purely abstract mathematical objects, but to actual physical processes, when we observe them (and indeed some of the most important constraints on information in our data arise from how we observe), so an explanation in terms of constraints in Lagrangians had better have a clear and fairly direct physical interpretation; fortunately it does.

 A natural interpretation of the Lagrangian constraints arises in thinking about the scaling at which information is gathered from the system by the process of observation and how this interacts with natural scales at which information is preserved in the organization of the observed system. Earlier publications, rooted in the maximum entropy concept, used the idea of convolution to describe how information could be preserved (or lost) as large numbers of random, small scale, processes interact and are observed. This line of thought appears to have led Frank to the realization that the organizing framework for the constraints is, at its core, a set of expressions describing patterns of invariance. Three types of invariance – shift and stretch (which combine to give affine invariance) and rotation, are sufficient to account for the fact that a few common probability patterns in biological and physical systems spanning many orders of magnitude, describe the majority of empirical datasets. Frank & Smith (2011) ^1^ gives a comprehensive early account of using the concept of symmetries (invariances) to derive many probability patterns. A recent strand of papers (of which the current one is an example) employ the concept of invariance to show how several specific types of biological pattern arise.

 In these papers, the concept of invariance is given primacy, and - explicitly in the current example - maximum entropy is viewed as a derived property that is not needed to explain the commonality of probability patterns. To reiterate an earlier point, quite aside from the technical merits, theoretical depth, and potential applications of the work itself, Frank’s publications on this topic are an interesting publication trail for those studying the development of theory in biology. They present an example of how one scientist’s thinking on a subject changes and develops over time. I’m laboring the description of the context for the work, because, as I will outline in what follows, I think the most important critique lies not in the technical details as they apply to species abundance distributions, but to the epistemic basis for the whole endeavor.

***Invariance and maximum entropy***

 Frank & Bascompte (F&B) argue that the log series for species abundance arises naturally when one considers two assumptions; first that there is affine invariance at the scale of proportional processes that act on species abundance (so, for example, birth and death), and second that there is a constraint on average abundance. Algebraic analysis, assuming a standard exponential form for the probability distribution, then shows that these two assumptions are sufficient to induce the log-series form; the probability distribution describing abundance. The authors contrast this analysis with the one owing to Harte, which is based on a maximum entropy interpretation. F&B note that working from first principles, assuming a constraint on average abundance, and starting from the assumption that entropy is maximized in the abundance distribution, one ends up with the exponential distribution as the maximum entropy form for species abundance. This is at odds with empirical observation.

 If we assume that the observed distribution of species abundances ***is*** a maximum entropy distribution, then this analysis tells us that simple constraint on the average abundance is insufficient to induce the observed probability pattern, which leads to three possible alternatives. First, some further constraint is required on the abundance distribution so that the derived form matches observation (this is essentially the approach taken by Harte). Second, the abundance distribution is itself dependent on one or more constraints in some other process (for which entropy is maximized) and the joint effect of these two sets of constraints results in the observed log series distribution (this is the approach taken by F&B). Thirdly, we abandon the premise that species abundance is a maximum entropy distribution and look for explanations in some other room in the library of all possible theories. The third option is a drastic one; especially when there are good arguments in related fields of research that support the idea that Nature does indeed confront us with maximum entropy distributions when we make observations. For example, in discussing the correspondence between entropy maximization and description length minimization, Grunwald (2007, p644) ^2^ writes:

 “ *we imagine a two-player game between Nature and Statistician. Nature first picks a distribution and then generates an outcome X according to it; Statistician picks a code and uses it to describe X. Nature is allowed to select any distribution she likes, as long as it satisfies E[ϕ(X)]=μ, and Statistician is allowed to use any code whatsoever. Nature’s goal is to maximize Statistician’s expected codelength, and Statistician’s goal is to minimize it. … the best (maximum codelength_ that she can achieve if she has to move first is equal to the best (minimum codelength) that Statistician can achieve if he has to move first. Surprisingly, under weak conditions both Nature’s maximin and Statistician’s minimax strategy turn out to be the Maxent distribution…*”

 There is, I believe, an important connection between MDL and Frank’s program of explanation for biological patterns and it is captured in the quotation from Grunwald’s (2007) ^2^ book given above. In a loose sense, one might cast Frank’s investigation of pattern as an inquiry into what Nature is doing in the game described by Grunwald. The conclusion is that she is playing a strategy of showing us maximum entropy distributions. As Grunwald (2007) ^2^ points out, this is the optimal conclusion for us to reach (playing the role of Statistician) if we want our adopted descriptions to be optimal in the sense of minimizing our expected maximum error. The Kraft-McMillan inequality establishes the correspondence between codelength functions and probability distributions, so Grunwald’s game between Nature and Statistician can be rephrased directly by substituting “probability distribution” for “codelength”. But, there is an additional, epistemic, connection between MDL and what Frank and his co-authors on this and other papers are doing.

 In establishing MDL Jorma Rissanen was attempting to establish a principle for model selection ***and*** inference that was free from the need for prior assumptions about the process (or model) generating the observed data. Here is the opening paragraph of Rissanen’s (1978) ^3^ paper on model selection:

*This study is an attempt to derive a criterion for estimation of both the integer-valued structure parameters and the real-valued parameters of dynamic systems starting from a single natural and fundamental principle: the least number of digits it takes to write down an observed sample of a time series*.

 Frank’s scheme, for describing why particular models ( *i.e.* probability distributions) describe is an alternative, but also hypothesis-free, attempt to describe what Nature is doing. It’s important to clarify what is meant in saying Frank’s approach is hypothesis-free. It is simply this. The method does not select a particular probability distribution (equivalently, a model or codelength function) for the data *a priori*, but instead establishes a few mechanistically motivated constraints on information, given the context of the data and the measuring process, and uses those to infer the form of the probability distribution one expects the data to follow. This idea of making well-motivated choices about the identity of the best description of observed data is also enshrined in MDL in the “luckiness principle” (see Grunwald (2007) ^2^ Ch14) further emphasizing the connection between the two lines of investigation.

 Why does this matter in relation to the paper by F&B? As I mentioned earlier the answer is more one of process and principle than technical detail. The importance of the current paper is that it adds to argument, advanced by Frank, that biological observations of all kinds can be systematized; general principles operate that allow us to form expectations about the distributional properties of our data in a non *ad hoc* manner. I applaud this effort and think that it’s a contribution to modern biology that will come to be seen as a major advance in the philosophical grounding of the subject, which is why I find the current paper somewhat frustrating. My main concern is this. In seeking to establish an invariance principle as taking precedence in some epistemiological sense, over the principle of maximum entropy, I think the authors make a mistake, and one that threatens the clarity of the preceding work by Frank and others on probability patterns. In essence the problem is that invariance and maximum entropy are not alternatives, the former is one of two approches commonly used to solve/understand maximum entropy problems, the other being the method of Lagrangian multipliers. I would characterize the conceptual shift in this paper (from Frank’s previous work) not as a shift from maximum entropy to invariance, but as a shift in focus on solutions to the maxent problems from approaches grounded in Lagrangians to approaches derived from the concepts of invariances, particularly symmetry groups. Both approaches are firmly within the overall framework of maximum entropy. So, my main request to the authors would be that they consider re-casting the paper along the lines just outlined and less as a demonstration that a principle of invariance supersedes the maximum entropy principle in describing biological patterns, in particular species abundance distributions.

 To anchor this argument more firmly to the paper (and draw in the MDL connection) here are a couple of points where I think the authors need to offer the reader a little more support for their proposal. F&B argue that their approach, based on invariance, offers a clearer rationale for deriving/explaining appropriate distributional forms than those based in either “maximum entropy” or mechanistic neutral theories. For example, in the section “Maximum entropy and the gamma lognormal” F&B note:

*By maximum entropy, all of the information in Hubbell’s mechanistic process theory of neutrality and the matching gamma-lognormal pattern reduces to maximum randomness subject to these three constraints*.

 However it is very unlikely that we would have derived the correct form by maximum entropy without knowing the answer in advance. This limitation emphasizes that maximum entropy provides deep insight into process and pattern, but often we need an external theory to guide our choice among various possible maximum entropy formulations.

 I would argue that subsequent derivation based on invariances is no less opaque, and someone attempting the derivation would, similarly, need to know where they were going in order to get there. It is of interest to point out that here, as in MDL, access to external theory will be of value in achieving results. This idea of externally motivated choice of approach is also apparent in the second example I would ask the authors to consider.

 In the discussion of the log series pattern, F&B contrast their approach, with that offered by Harte. As we already noted, F&B point out that starting from the canonical form for probability distributions, a constraint on the expected value for the observations leads to the exponential distribution as the emergent form; a result that demonstrably fails to deal with observed species abundance data. Harte solved this problem by introducing a second variable that is similarly constrained at the same scale as the expectation of abundance. F&B criticize Harte’s approach, in essence, on the grounds that it is an *ad hoc* solution, arguing that their approach, in which constraints (invariances) are placed on underlying demographic processes, has a clearer rationale. While F&B’s argument is persuasive, I think it needs to be strengthened and somewhat expanded. Here’s why:

 As a I pointed out above, failure of the simple constraint on average abundance to lead to the log series requires us to come up with an alternative hypothesis for why the log series is observed. In MDL terms, we need a better description of the data. From a logical perspective, there doesn’t appear to be any difference between adding an assumption of a variable at the same scale as abundance being under constraint, and an assumption of proportional demographic processes at a lower scale being constrained. In fact, from a model parsimony (MDL) perspective, a model that relies on adding a whole additional scale of processes may be viewed as propagating unnecessary complication. Furthermore, it doesn’t seem like too much of a stretch to suggest that constraints on proportional processes at a lower scale, might not give rise to quantity at the same scale as the expectation of abundance that is similarly constrained, when measured at that scale? Is the issue simply one of how phenomenological one likes one’s models to be? And if so, what guidance can be given to those who want to pursue the type of analysis proposed by F&B? As a first stab at an answer to that question would something along the following lines offer budding invariance analysts a template to work from?

**I**dentify the **O**bjects that are the subject of your interest (SAD’s in the current case). **U**se **O**ccam’s **R**azor and **M**ethodological **I**ndividualism, when deciding where to look for constraints. (IOUORMI “I owe you, or me”). The combination of Occam’s Razor and Methodological Individualism should guide investigators to look for the simplest model built from processes operating at one level below the objects of interest in the organizational hierarchy of the systems of interest.

 In any case, I applaud the authors on showing that the diversity of known SAD data can be organized and explained by a unified principle, that has a clear theoretical and physical basis.

I confirm that I have read this submission and believe that I have an appropriate level of expertise to confirm that it is of an acceptable scientific standard.

## References

[1] : A simple derivation and classification of common probability distributions based on information symmetry and measurement scale. *J Evol Biol*.2011;24(3) : 10.1111/j.1420-9101.2010.02204.x 469-84 10.1111/j.1420-9101.2010.02204.x 21265914

[2] : The Minimum Description Length Principle. *The MIT Press, Cambridge, MA*.2007;

[3] : Modeling by shortest data description. *Automatica*.1978;14(5) : 10.1016/0005-1098(78)90005-5 465-471 10.1016/0005-1098(78)90005-5

# Peer review: Reviewer response for version 1

**DOI:** 10.5256/f1000research.23789.r58055

**Published:** 2020-01-24

**Authors:** David Alonso

**Affiliations:** 1 Theoretical and Computational Ecology, Center for Advanced Studies of Blanes (CEAB-CSIC), Blanes, Spain

This contribution emphasizes how the concept of invariance connects patterns to processes leading to an original synthesis of previous approaches. In particular, this piece of work illustrates the application of the concept of invariance to deduce the most commonly observed species abundance distributions (SAD) in nature. The authors succeed in giving a general overview of the relation between invariance, process, and pattern.

 As an illustration, they present two key theoretical results. First, they derive the log series SAD from a simple maximum entropy argument. When species abundances fluctuate randomly on a log scale, this is, process values 'r' are random (where r is defined as log n), and these fluctuations are only subject to an ecological constraint limiting total abundance, then the log series distribution, initially introduced by R. A. Fisher, naturally arises. Second, they show that Hubbell's neutral model is the simple expression of three basic invariances, which correspond to a maximum entropy model constraining average species abundances, and the average and variance of the demographic processes influencing abundance. 

 The first theoretical result is far from original as the same argument is clearly presented by Pueyo *et al.*(2007) ^1^ in "The maximum entropy formalism and the idiosyncratic theory of biodiversity" where clearly, after a scale invariance argument is done, the log series again naturally arises from a maximum entropy derivation by only constraining on total abundance. However, the second result and the whole emphasis on the generality and the power of the invariance approach in ecology is a true novel contribution to the field. 

 The authors state, in their conclusions, that "the invariance approach is the only way to relate process to pattern". The authors emphasize that, in order to uncover the plausible underlying mechanisms underlying an observed pattern, one needs first to pay special attention to the general invariant form of probability patterns. In the future, I would like to see the authors' invariance approach to apply, more generally to other patterns in ecology.

I confirm that I have read this submission and believe that I have an appropriate level of expertise to confirm that it is of an acceptable scientific standard.

## References

[1] : The maximum entropy formalism and the idiosyncratic theory of biodiversity. *Ecol Lett*.2007;10(11) : 10.1111/j.1461-0248.2007.01096.x 1017-28 10.1111/j.1461-0248.2007.01096.x 17692099PMC2121135
